# The long-term effects of the Covid-19 infection on cardiac symptoms

**DOI:** 10.1186/s12872-023-03322-8

**Published:** 2023-06-06

**Authors:** Reza Golchin Vafa, Reza Heydarzadeh, Mohammadhossein Rahmani, Ali Tavan, Soroush Khoshnoud Mansorkhani, Bardia Zamiri, Farhang Amiri, Alireza Azadian, Amin Khademolhosseini, Mohammad Montaseri, Nazanin Hosseini, Seyed Ali Hosseini, Javad Kojuri

**Affiliations:** 1 Cardiology Department, Shiraz University of Medical Sciences, Shiraz, Iran; 2 Professor Kojuri Cardiology Clinic, Niayesh St. Niayesh Medical Complex, Shiraz, Iran; 3 Clinical Education Research Center, Shiraz University of Medical Sciences, Shiraz, Iran

**Keywords:** COVID-19, Post-Acute COVID-19 syndrome, Cardiovascular system, Signs and symptoms

## Abstract

**Background:**

Besides the lungs, coronavirus disease 2019 (COVID-19) can affect the cardiovascular, digestive, urinary, hepatic, and central nervous systems. Other than its short-term effects, COVID-19 may also cause long-term complications. In this study, we assessed long-term COVID-19 cardiovascular symptoms among patients in a cardiovascular clinic.

**Method:**

A retrospective cohort was conducted between October 2020 to May 2021 on patients at an outpatient cardiovascular clinic in Shiraz, Iran. Patients with a history of COVID-19 at least one year before their referral were included. Baseline information was extracted from the clinic’s database. Data were collected regarding symptoms like dyspnea, chest pain, fatigue, and palpitations after a year of COVID-19. We also noted any major adverse cardiac events (MACE).

**Results:**

Most common symptoms after a year of COVID-19 were exertional dyspnea (51.2%), dyspnea at rest (41.6%), fatigue (39%), and chest pain (27.1%). The symptoms were more prevalent in hospitalized patients than in non-hospitalized patients. The prevalence of MACE was about 6.1% during the 12-month follow-up, with this rate being higher in those with a history of hospitalization or comorbid diseases.

**Conclusion:**

The prevalence of cardiovascular symptoms was fairly high in patients at our clinic a year after COVID-19, and the most common symptom was dyspnea. Hospitalized patients had more MACE. (Clinicaltrial.gov number: NCT05715879)(04/02/2023).

## Introduction

The coronavirus disease 2019 (COVID-19) pandemic has affected all aspects of life, including the health system and education [1–5]. The agent responsible, severe acute respiratory syndrome coronavirus-2 (SARS-CoV-2), is a single-stranded RNA β-coronavirus believed to invade human cells via the angiotensin-converting enzyme 2 (ACE2) receptor. Hence, despite primarily affecting the pulmonary system, COVID-19 can damage other organ systems that express ACE2 receptors [6, 7]. Importantly, COVID-19 causes the release of reactive oxygen species that contribute to cell death. Reactive oxygen species release factors such as nuclear factor kappa B (NF-κB) can trigger a cytokine storm. Moreover, COVID-19 also induces the cytokine storm via the release of inflammatory factors by immune and non-immune cells [8, 9]. Through multiple mechanisms, COVID-19 can affect the pulmonary, cardiovascular, digestive, urinary, hepatic, and central nervous systems [10].

Cardiovascular diseases (CVDs) are one of the most critical factors affecting the morbidity and mortality caused by COVID-19. Coronary artery disease (CAD), heart failure (HF), hypertension (HTN), and arrhythmias lead to a higher mortality rate in COVID-19 patients [11]. On the other hand, COVID-19 causes cardiovascular complications that can be fatal, including myocarditis, myocardial infarction, acute heart failure, arrhythmia, and venous thromboembolism [12]. Therefore, it seems there is a mutual relationship between COVID-19 and CVDs.

Recent studies have found that COVID-19 has long-term effects on general health besides its short-term complications. The long-term complications of COVID-19 involve the cardiovascular, pulmonary, central nervous, renal, and gastrointestinal systems [13]. A meta-analysis indicated that 80% of COVID-19 patients remained symptomatic after two weeks past the beginning of the infection. The most common symptoms were dyspnea, fatigue, headache, hair loss, and attention deficit disorders. The study found that COVID-19 had more than fifty long-term consequences on patients’ health [14]. Continuation of COVID-19 signs and symptoms for more than four weeks is known as ‘prolonged COVID-19’, including ‘ongoing symptomatic COVID-19’ (having symptoms for 4 to 12 weeks) and ‘post-COVID-19 syndrome’ (experiencing symptoms for more than 12 weeks) [15].

Prolonged COVID-19 can cause disorders in cardiac rhythm, including supraventricular and ventricular tachycardias, atrial fibrillation, and even complete heart block; similarly, cardiovascular complications were seen in the long-term follow-up of SARS patients [16, 17]. Even among those not experiencing acute coronary syndrome (ACS), myocardial injury is thought to not be uncommon, with possible long-term effects [18]. Davis and colleagues reported that cardiovascular symptoms such as palpitations (68%), chest pain (53%), and fainting (13%) were detected in up to 86% of patients with prolonged COVID-19 after seven months [19]. Cardiovascular risk factors may also be affected since the previous SARS outbreak was linked with hyperlipidemia and glucose intolerance in the long term [20].

In the existing literature, there is little evidence about the long-term effects of COVID-19 on cardiovascular symptoms after a 12-month follow-up. Hence, this study assessed cardiovascular symptoms and complications 12 months after COVID-19 in patients at a cardiovascular clinic.

## Methods

This retrospective cohort study was conducted between October 2020 to May 2021. The study population was patients referring to Professor Kojuri Cardiovascular Clinic in Shiraz, Iran (email: kojurij@yahoo.com, webpage: http://kojuriclinic.com). A database of patients' information is kept at the clinic, including data on underlying diseases, signs and symptoms, medications, laboratory tests, electrocardiography, and echocardiography. Expert cardiologists document the data on every visit. Most patients are healthy individuals who visit the clinic for check-ups, though some have a cardiovascular condition.

The inclusion criteria were having a history of COVID-19 confirmed by polymerase chain reaction (PCR) or suggested by High-Resolution Computed Tomography (HRCT) findings at least one year ago. The exclusion criteria were having a history of documented COVID-19 less than a year ago or having a probable history of COVID-19 not confirmed by PCR or HRCT. Patients with incomplete data before COVID-19 or with outdated data were also excluded. The data had a time interval of more than one month before COVID-19, or one month after one year of recovery from COVID-19, are considered outdated data.

We contacted the enrolled patients by telephone to get informed about their symptoms, such as dyspnea at rest, dyspnea on exertion (DOE), orthopnea, paroxysmal nocturnal dyspnea (PND) [21], chest pain (CP) [22], fatigue [23], and palpitations [24]. Patients were asked to rate their dyspnea at rest from 0 to 10, according to the 10-category ratio. A score of zero means no breathing discomfort, and ten indicates the most severe dyspnea. A score between 1 to 4 is considered mild, 5 to 6 moderate, and 7 to 10 severe. We also used functional classes 1 to 4 to assess dyspnea. Functional class 1 means no limitations in daily activities; class 2 means mild exertional dyspnea; class 3 indicates moderate dyspnea with daily activities; and class 4 denotes dyspnea at rest [25]. Chest pain was defined in line with the American Heart Association’s classification system as “non-cardiac,” “possible cardiac,” or “cardiac” [22].

A history of major adverse cardiovascular events (MACE) during the year after COVID-19 and admission due to COVID-19 were also noted. MACE is defined as myocardial infarction (MI), admission due to heart failure (HF), stroke, cardiac death, and revascularization procedures, including a coronary artery bypass graft (CABG) or percutaneous coronary intervention (PCI) [26].

Hypertension was defined as a clinical systolic blood pressure ≥ 140 mm Hg or diastolic blood pressure ≥ 90 mm Hg on repeated assessments [27]. Diabetes mellitus (DM) was diagnosed based on the 2020 American Diabetes Association (ADA) guidelines [28]. Dyslipidemia was defined as abnormalities in triglycerides (TG; > 150 mg/dL), low-density lipoprotein (LDL) cholesterol (> 100 mg/dL), or high-density lipoprotein (HDL) cholesterol (< 40 mg/dL) [29]. Current smokers were defined as those who had smoked ≥ 100 cigarettes and had smoked during the 30 days preceding the study. Former smokers had stopped smoking more than 30 days before the research [30].

We extracted data patients’ information before COVID-19 from the clinic’s database. This included baseline demographic data, COVID-19 vaccination history, HTN, dyslipidemia or hyperlipidemia (HLP), DM, smoking, CVD, and prescribed medications.

Statistical analysis was performed using SPSS for Windows ver. 26 (IBM Corp., Armonk, NY, USA). We described continuous variables by mean ± standard deviation. Categorical variables were described by frequency and percentage. We used repeated measure ANOVA and the paired-sample t-test for normal distribution variables and Wilcoxon’s signed-rank test for repeated categorical variables. Pearson's chi-squared and Kruskal–Wallis tests were applied to categorical data. We controlled the effects of confounding factors by using generalized linear models and repeating analyses in different subgroups. We estimated the minimum sample size by [*n* = (Z2 × P × (1-P))/e2], considering the 95% confidence interval and 50% prevalence of the symptoms. The minimum sample size was 386 patients. Statistical significance was indicated when *P* < 0.05.

All patients were informed about the details of this research and provided their informed consent.. For informed consent, we contacted the patients by telephone and described the detail of the study and the anonymity of their data. Patients who declined to participate were excluded. The study protocol was approved by the Ethics Committee of Shiraz University of Medical Sciences under code IR.SUMS.MED.REC.1401.465. All methods were performed in accordance with the Helsinki guidelines and regulations.

## Results

### Demographic

There were 879 eligible patients for this study. We excluded 36 patients because of incomplete data, inaccessibility, and lack of consent to answer the questions by telephone. Finally, the study was conducted on 843 patients; 449 women (53.4%) and 394 (46.7%) men. The mean age of the participants was 57.2 ± 13.6 years.

The number of patients with a previous revascularization history was 229 (≈27.2%), including 183 post-PCI patients (≈21.7%), 25 post-CABG patients (≈3%), and 21 patients with a history of both PCI and CABG (≈2.5%). The prevalence of underlying diseases was 62.2% for HTN, 27.2% for DM, 24.9% for dyslipidemia, and 5.6% for HF. In this study, 48 people (5.7%) were current cigarette smokers, and 47 (5.6%) were previous cigarette smokers.

At the beginning of the study, only 50 patients (5.9%) had received two doses of COVID-19 vaccination, and 38 people (4.5%) had been vaccinated with the first dose. Totally, 154 patients (18.3%) were admitted to a hospital due to COVID-19. The mean duration of the days after COVID-19 was about 383 ± 19 days.

### Dyspnea

The prevalence of dyspnea a year after COVID-19 was about 41.6% (351 patients); the frequency of mild, moderate, and severe dyspnea was estimated at 254 (30.1%), 56 (6.6%), and 41 (4.9%), respectively. The prevalence of different functional classes was I (411, 48.8%), II (348, 41.3%), III (47, 5.6%), and IV (37, 4.4%). Patients with a history of admission due to COVID-19 had a higher functional class than outpatients (*P* = 0.001). Women had more activity limitations (considering their functional class) than men after a year of COVID-19 recovery (*P* = 0.003) (Table 1).

**Table 1:** The prevalence of different functional classes of dyspnea after a year of COVID-19 recovery in different subgroups

Variable	I	II	III	IV	*P*-value
Hospital admission due to COVID-19, n (%)					
No	348 (50.5)	283 (41.1)	36 (5.2)	22 (3.2)	**0.001**
Yes	63 (40.9)	65 (42.2)	11 (7.1)	15 (9.7)	
Sex, n (%)					
Male	208 (52.8)	160 (40.6)	13 (3.3)	13 (3.3)	**0.003**
Female	203 (45.2)	188 (41.9)	34 (7.6)	24 (5.3)	
Age, n (%)					
15 to 30 years	15 (57.6)	9 (34.6)	2(7.6)	0	0.325
30 to 45 years	80 (52.3)	61 (39.9)	8 (5.2)	4 (2.6)	
45 to 60 years	120 (43.2)	127 (45.7)	18 (6.5)	13 (4.7)	
60 to 75 years	165 (51.9)	124 (39)	12 (3.8)	17 (5.3)	
> 75 years	31 (45.6)	27 (39.7)	7 (10.3)	3 (4.4)	
Hypertension, n (%)					
Yes	251 (47.9)	222 (42.4)	27 (5.2)	24 (4.6)	0.696
No	160 (50.2)	126 (39.5)	20 (6.3)	13 (4.1)	
Diabetes mellitus, n (%)					
Yes	111 (48.5)	94 (41)	14 (6.1)	10 (4.4)	0.859
No	300 (48.9)	254 (41.4)	33 (5.4)	27 (4.4)	
Smoking, n (%)					
Previous smoker	22 (46.8)	22 (46.8)	2 (4.3)	1 (2.7)	0.943
Current smoker	22 (45.8)	21 (43.8)	4 (8.3)	1 (2.1)	
Non-smoker	367 (49.1)	305 (40.8)	41 (5.5)	35 (4.7)	
Hyperlipidemia, n (%)					
Yes	109 (51.9)	87 (41.4)	7 (3.3)	7 (3.3)	0.106
No	302 (47.7)	261 (41.2)	40 (6.3)	30 (4.7)	
Coronary artery disease, n (%)					
Yes	106 (46.3)	95 (41.5)	16 (7)	12 (5.2)	0.202
No	305 (49.7)	253 (41.2)	31 (5)	25 (4.1)	

At the end of the study, 407 patients (48.3%) reported no differences in regard to their dyspnea before and after a year following COVID-19. On the other hand, 157 patients (18.6%) reported worsening of their dyspnea, and interestingly, dyspnea was alleviated in 279 patients (33.1%). There were no differences between dyspnea scores before and after a year of COVID-19 based on the 10-score scale (*P* = 0.408). There was also no significant difference between the severity of dyspnea before and after a year of COVID-19 (*P* = 0.494).

Among the admitted ones due to COVID-19, 76 patients (49.4%) reported no differences between their dyspnea before and after a year following COVID-19; however, 55 patients (35.7%) reported that their dyspnea worsened after a year following COVID-19, and 23 patients (14.9%) reported improvement of their symptoms (*P* < 0.001). On the other hand, among patients not admitted due to COVID-19, 331 participants (48%) reported no differences between their baseline and follow-up dyspnea. However, 256 patients (37.2%) reported alleviation of their dyspnea, and 102 patients (14.8%) reported worsening of their symptoms (*P* < 0.001) (Table 2).

**Table 2:** The change in dyspnea between baseline and follow-up in different subgroups

Variable	No change	Worsened	Improved	*P*-value
Hospital admission due to COVID-19, n (%)				
No	331 (48.0)	102 (14.8)	256 (37.2)	** < 0.001**
Yes	76 (49.4)	55 (35.7)	23 (14.9)	
Sex, n (%)				
Male	190 (48.2)	74 (18.8)	130 (33)	0.994
Female	217 (48.3)	83 (18.5)	149 (33.2)	
Age, n (%)				
15 to 30 years	12 (46.1)	4 (15.3)	10 (38.4)	0.130
30 to 45 years	69 (45.1)	25 (16.3)	59 (38.6)	
45 to 60 years	121 (43.5)	55 (19.8)	102 (36.7)	
60 to 75 years	175 (55)	56 (17.6)	82 (27.4)	
> 75 years	30 (44.1)	17 (25)	21 (30.9)	
Hypertension, n (%)				
Yes	269 (51.3)	157 (18.6)	279 (33.1)	**0.013**
No	138 (43.3)	56 (17.6)	125 (39.2)	
Diabetes mellitus, n (%)				
Yes	112 (48.9)	42 (18.3)	75 (32.8)	0.975
No	295 (48)	115 (18.7)	204 (33.2)	
Smoking, n (%)				
Previous smoker	20 (42.6)	10 (21.3)	17 (36.2)	0.438
Current smoker	29 (60.4)	8 (16.7)	11 (22.9)	
Non-smoker	358 (47.9%)	139 (18.6)	251 (33.6)	
Hyperlipidemia, n (%)				
Yes	109 (51.9)	38 (18.1)	63 (30)	0.446
No	298 (47.1)	119 (18.8)	216 (34.1)	
Coronary artery disease, n (%)				
Yes	100 (43.7)	55 (24)	74 (32.3)	**0.042**
No	307 (50)	102 (16.6)	205 (33.4)	

In hypertensive patients, the prevalence of dyspnea development was significantly higher than in non-hypertensives (*P* = 0.013). Patients with CAD experienced more worsening dyspnea than patients without CAD (*P* = 0.042). There was no association between dyspnea differences and any other cardiovascular risk factors, including age (*P* = 0.130), DM (*P* = 0.975), smoking (*P* = 0.438), and HLP (*P* = 0.446) (Table 2).

### Dyspnea on exertion, orthopnea, and paroxysmal nocturnal dyspnea

A year after COVID-19, 432 patients (51.2%) reported suffering from exertional dyspnea. Among patients admitted to the hospital, more cases (91 patients, 21.1%) reported DOE compared with those managed in outpatient settings (63 patients, 15.3%) (*P* = 0.033). Women reported more DOE than men (*P* = 0.032). There was also no association between DOE and any of the cardiovascular risk factors, including age (*P* = 0.197), HTN (*P* = 0.570), DM (*P* = 0.938), smoking (*P* = 0.876), HLP (*P* = 0.301), and CAD (*P* = 0.395) (Table 3).

**Table 3:** The prevalence of dyspnea on exertion, orthopnea, and paroxysmal nocturnal dyspnea in different subgroups

Variable	DOE	*P*-value	Orthopnea	*P*-value	PND	*P*-value
Hospital admission due to COVID-19, n (%)						
Yes	91 (59.1)	**0.033**	26 (16.9)	**0.004**	24 (15.6)	0.610
No	341 (49.5)		59 (8.6)		96 (13.9)	
Sex, n (%)						
Male	186 (47.2)	**0.032**	29 (7.4)	**0.016**	54 (13.7)	0.694
Female	246 (54.8%)		56 (12.5)		66 (14.7)	
Age, n (%)						
15 to 30 years	11 (44.0)	0.197	2 (8)	0.880	5 (20)	0.305
30 to 45 years	73 (47.7)		12 (7.8)		23 (15)	
45 to 60 years	158 (56.8)		29 (10.4)		48 (17.3)	
60 to 75 years	153 (48.1)		36 (11.3)		38 (11.9)	
> 75 years	37 (54.4)		6 (8.8)		6 (8.8)	
Hypertension, n (%)						
Yes	273 (52.1)	0.57	55 (10.5)	0.639	71 (13.5)	0.478
No	159 (49.8)		30 (9.4)		49 (15.4)	
Diabetes mellitus, n (%)						
Yes	118 (51.5)	0.938	25 (10.9)	0.609	31 (13.5)	0.825
No	314 (51.1)		60 (9.8)		89 (14.5)	
Smoking, n (%)						
Previous smoker	25 (53.2)	0.876	1 (2.1)	**0.058**	2 (4.3)	0.063
Current smoker	26 (54.2)		7 (14.6)		6 (12.5)	
Non-smoker	381 (50.9%)		77 (10.3)		112 (15)	
Hyperlipidemia, n (%)						
Yes	101 (48.1)	0.301	22 (10.5%)	0.793	32 (15.2)	0.649
No	331 (52.3)		63 (10)		88 (13.9)	
Coronary artery disease, n (%)						
Yes	123 (53.7)	0.395	29 (12.7%)	0.156	37 (16.2)	0.321
No	309 (50.3)		56 (9.1)		83 (13.5)	

*DOE* Dyspnea on exertion, *PND* Paroxysmal nocturnal dyspnea

After a year, 85 patients (10.1%) reported orthopnea. Among hospitalized patients, 26 patients (16.9%) had orthopnea, and among outpatient ones, 59 cases (8.6%) suffered from orthopnea after a year following COVID-19 (*P* = 0.004). The prevalence of orthopnea among women (56, 12.5%) was higher in comparison to men (29, 7.4%) (*P* = 0.016). There was no association between orthopnea and any of the cardiovascular risk factors, including age (*P* = 0.88), HTN (*P* = 0.639), DM (*P* = 0.609), smoking (*P* = 0.111), HLP (*P* = 0.793), and CAD (*P* = 0.156) (Table 3).

The prevalence of PND was 14.2% (120 patients) after one year following the infection. There was no meaningful association between PND and hospitalization (*P* = 0.610), gender (*P* = 0.694), age (*P* = 0.305), HTN (*P* = 0.478), DM (*P* = 0.825), smoking (*P* = 0.117), HLP (*P* = 0.649), and CAD (*P* = 0.321) (Table 3).

### Chest pain, fatigue, and palpitations

After one year, the prevalence of CP was 229 (27.1%). Among these, 66 patients (7.8%) reported cardiac CP, 163 patients (19.3%) had non-cardiac CP, and 614 cases (72.8%) reported no CP. The prevalence of cardiac and non-cardiac CP among hospitalized patients was 16.2% (25 patients) and 24.7% (38 patients), respectively. Among non-hospitalized patients, 41 (6%) reported cardiac CP, and 125 patients (18.1%) had non-cardiac CP (*P* < 0.001). There was no association between CP and sex (*P* = 0.875), age (*P* = 0.749), HTN (*P* = 0.176), DM (*P* = 0.702), smoking (*P* = 0.198), HLP (*P* = 0.865), and CAD (*P* = 0.233) (Table 4).

**Table 4:** The prevalence of chest pain, fatigue, palpitations, and major coronary adverse events in different subgroups

Different subgroups	Non-cardiac CP	Cardiac CP	*P*-value	Fatigue	*P*-value	Palpitations	*P*-value	MACE	*P*-value
Hospital admission due to COVID-19, n (%)									
Yes	28 (24.7)	25 (16.2)	** < 0.001**	60 (39)	1	19 (12.3)	0.567	20 (13)	**0.001**
No	125 (18.1)	41 (6%)		269 (39)		73 (10.6)		34 (4.9)	
Sex, n (%)									
Male	79 (20.1)	30 (7.6)	0.876	142 (36)	0.104	51 (12.9)	0.078	26 (6.6)	0.470
Female	84 (18.7)	36 (8)		187 (41.5)		41 (9.1)		29 (6.2)	
Age, n (%)									
15 to 30 years	9 (36)	1 (4)	0.749	8 (32)	0.099	5 (20)	0.351	1 (4)	0.651
30 to 45 years	27 (17.6)	12 (7.8)		49 (32)		20 (13.1)		8 (5.2)	
45 to 60 years	57 (20.5)	23 (8.3)		117 (42.1)		24 (8.6)		14 (5)	
60 to 75 years	58 (18.2)	23 (7.2)		133 (41.8)		33 (10.4)		25 (7.9)	
> 75 years	12 (17.6)	7 (10.3)		21 (30.9)		10 (14.7)		6 (8.8)	
Hypertension, n (%)									
Yes	110 (21)	44 (8.4)	0.176	209 (39.9)	0.560	51 (9.7)	0.172	46 (8.8)	** < 0.001**
No	53 (16.6)	22 (6.9)		120 (37.6)		41 (12.9)		8 (2.5)	
Diabetes mellitus, n (%)									
Yes	48 (21)	19 (8.3)	0.702	93 (40.6)	0.579	29 (12.7)	0.322	18 (7.9)	0.342
No	115 (18.7)	47 (7.7)		236 (38.4)		63 (10.3)		36 (5.9)	
Smoking, n (%)									
Previous smoker	10 (21.3)	4 (8.5)	0.198	13 (27.7)	0.259	5 (10.6)	0.935	5 (10.6)	**0.049**
Current smoker	7 (14.6)	8 (16.7)		19 (39.6)		6 (12.5)		7 (14.6)	
Non-smoker	146 (19.5)	54 (7.2)		297 (39.7)		81 (10.8)		42 (5.6)	
Hyperlipidemia, n (%)									
Yes	43 (20.5)	17 (8.1)	0.865	89 (42.4)	0.254	31 (14.8%)	**0.042**	23 (11%)	**0.003**
No	120 (19)	49 (7.7)		240 (37.9)		61 (9.6)		31 (4.9%)	
Coronary artery disease, n (%)									
Yes	51 (22.3)	21 (9.2)	0.233	83 (36.2)	0.341	27 (11.8)	0.621	25 (10.9)	**0.002**
No	112 (18.2)	45 (7.3)		246 (40.1)		65 (10.6)		29 (4.7)	

*CP* Chest pain, *MACE* Major adverse cardiac events

The prevalence of fatigue and palpitations was about 329 (39%) and 92 (10.9%) after a year of COVID-19. Patients with hyperlipidemia had more palpitations than others (*P* = 0.04). There was no association between fatigue or palpitations and hospitalization, sex, age, HTN, DM, smoking, and CAD (Table 4).

### Major adverse cardiac events

After a one-year follow-up of the COVID-19 patients, 54 cases (6.4%) showed MACE, including 44 ACS (5.2%), five hospitalizations due to heart failure (0.6%), four cerebrovascular accidents (0.5%), and one cardiac death (0.1%). The prevalence of MACE was higher in hospitalized patients (20, 13%) than in outpatients (34, 4.9%) (*P* = 0.001). Hypertensive cases had more incidence of MACE after a year of COVID-19 than non-hypertensives (*P* < 0.001). In patients with hyperlipidemia, 23 people (11%) reported MACE, while the prevalence of MACE in non-hyperlipidemic patients was 31 (4.9%) (*P* = 0.003). Patients with a history of previous revascularization had more MACE (*P* = 0.002). The prevalence of MACE in current and former smokers was 7 (14.6%) and 5 (10.6%). Although, 42 non-smokers (5.6%) had MACE after a year of COVID-19 (*P* = 0.049) (Table 4).

## Discussion

Dyspnea, as an important cardiovascular symptom, was reported by our study to be significantly common after COVID-19 (41.6%), even after one year. We found that in the subgroup of patients with more advanced diseases who needed hospitalization, worsening of dyspnea was more frequently reported compared to those not admitted to a hospital. The prevalence of worsening dyspnea was also higher in patients with CAD than in non-CAD patients. The frequency of DOE and orthopnea was higher in women than in men. Because dyspnea has different sources, we also used the NYHA classification to determine the severity of dyspnea, more specifically in cardiovascular settings. Women and hospitalized patients had more severe symptoms after a year of COVID-19 recovery, based on the NYHA. One study showed that about 43% of patients reported suffering from dyspnea two months after COVID-19 [31]. On the other hand, a meta-analysis by Alkodaymi et. Al showed that despite the heterogenicity of studies, the frequency of dyspnea was about 25% at three to six months but reached 31% when following patients for more than 12 months [32].

Mendola M et al. conducted a study on healthcare workers admitted due to COVID-19 to assess long-term symptoms. The most common symptom after recovery from COVID-19 was exertional and resting dyspnea. After ten months, the symptoms decreased significantly; however, mental problems continued [33]. Comelli et al. found that exertional dyspnea was the most common symptom after 12 months following COVID-19. Their study showed that comorbidities and being female were associated with more sequelae after 12 months from COVID-19 hospital admission [34]. Other studies also confirmed that comorbidities and the female gender have a significant role in experiencing more long-term symptoms. The differences may be due to various biologic responses or different patterns of receptors that the virus uses to enter the body in females and males [35–37]. Older patients and those with underlying lung disease or longer hospital courses are more prone to have fibrotic changes in their lungs after COVID-19. These changes lead to experiencing more pulmonary symptoms after a long-term follow-up [38].

Chest pain (CP) is the cardinal manifestation of cardiac and respiratory diseases. It also has various non-cardiopulmonary sources, such as chest wall syndrome, psychogenic etiologies, esophageal conditions, and other gastrointestinal disorders [39]. Although not negligible, when compared to dyspnea, CP was less frequently observed (27.7%) among patients in our study. Notably, the relative frequency of cardiac CP was much higher (*P* < 0.001) in hospitalized patients than in those not needing admission due to COVID-19. A previous study estimated the prevalence of CP at 20% in patients two months after COVID-19 [40]. Davis et al. showed that 53% of 3,762 patients experience CP after a 7-month follow-up from the infection [19]. Huang and colleagues demonstrated that CP continued in 7% of patients after a year following COVID-19 [41]. Furthermore, along with dyspnea and fatigue, CP is another component of the so-called “long COVID-19”, which may occur in 4.7–80% of cases, according to a systematic review of 25 observational studies [42]. In another review of 69 studies about post-COVID-19 complications, cardiac symptoms such as CP and palpitations were reported as common post-cure complications [43].

Long-term cardiovascular symptoms such as CP result from increased myocardial demand and inflammatory reactions, as seen in severe cases [44, 45]. Dani and colleagues figured that long-term symptoms, including CP, result from instability in the autonomic nervous system due to destruction by the virus or immune response [46]. In one study, approximately 78% of patients suffering from long-term symptoms had an abnormal cardiac MRI [47]. Hence, cardiovascular symptoms in prolonged COVID-19 should not be underestimated.

We figured that fatigue is among the most common symptoms that persist after 12 months of COVID-19. A cross-sectional study illustrated that about 93% of patients had fatigue three months after COVID-19. They also found that fatigue had no association with the severity of the disease [48]. Ansey et al. showed that approximately 60% of patients suffered from fatigue during a 12-month follow-up [49]. Studies revealed no associations between the severity of COVID-19, inflammatory markers, and fatigue. Although, women and patients with mental diseases more frequently experience fatigue [50]. Finally, it seems that different mechanisms and factors have roles in the presence of fatigue in the long-term [51].

Shechter et al. showed that the prevalence of MACE was zero after a 12-month follow-up [52]. The incidence of MACE in patients following COVID-19 may be predictable by biomarkers such as troponin [53]. Considerably, we found that about 6% of our patients developed MACE after COVID-19 in a one-year follow-up. Although cardiovascular complications of COVID-19 are well described [54], data about late cardiac events are lacking. Presumed pathogenesis consists of direct and indirect injury (immune and thromboembolism-mediated) [55]. We showed that patients with comorbid diseases, including HTN, smoking, HLP, and CAD, had a greater risk of developing MACE. Moreover, hospitalized patients were more prone to developing MACE than those not hospitalized. As a result, it’s necessary to follow COVID-19 patients with a history of hospital admission and comorbidities more precisely for evaluating cardiovascular complications.

Our study demonstrated that cardiovascular symptoms, including dyspnea, orthopnea, DOE, PND, and CP, are not uncommon among patients even after a long period following COVID-19. As noted previously, COVID-19 has considerable effects on the cardiovascular system besides the well-known respiratory problems. As a presumed mechanism, TGF-β1, angiotensin II, and other cytokines, which are elevated because of the severe inflammation evoked by COVID-19, are thought to affect cardiac myofibroblast differentiation, leading to fibrosis [56]. Moreover, vascular endothelium may be a target for the virus itself. Small and large vessels are damaged through several mechanisms leading to various presentations, including ACS, stroke, venous thromboembolism, and even limb ischemia. Altogether, regular follow-up of patients with COVID-19, considering the long-term consequences, is of great importance. Rehabilitation could help patients with prolonged COVID-19 relieve their symptoms, including fatigue and dyspnea, and improve their quality of life [57]. 

### Study limitations

The limitations of this study included its reliance on retrospective data collected via telephone interviews, which may lead to recall bias. However, the effects of such bias were minimized by the large sample size and the use of the clinic database. Another limitation was the fact that patients were selected from those regularly attending the clinic (mostly for check-ups), and there was no control group for the COVID-19 patients. Hence, the findings must be interpreted with caution. Further studies should assess long-term COVID-19 cardiovascular complications in different populations, considering the documented effects of this viral disease on cardiac functions.

## Conclusions

We showed that the prevalence of cardiovascular symptoms in COVID-19 patients was significantly high, even after a long-term follow-up. The most common symptoms include exertional dyspnea, dyspnea on rest, and fatigue. However, it should be noted that these symptoms may have other origins. The symptoms were more prevalent in hospitalized patients than in non-hospitalized. The prevalence of cardiovascular symptoms was higher in patients with comorbid diseases. Also, cardiovascular symptoms and major adverse cardiac events were more prevalent in hospitalized patients than in non-hospitalized ones.

## Data Availability

All data are available in professor Kojuri cardiology clinic, registry and available with reasonable request.

## Abbreviations

ACE2: Angiotensin-converting enzyme 2
ACS: Acute coronary syndrome
SARS-CoV-2: Severe Acute Respiratory Syndrome Corona Virus 2
CABG: Coronary artery bypass graft
CAD: Coronary artery disease
CP: Chest pain
CVD: Cardiovascular diseases
DM: Diabetes mellitus
DOE: Dyspnea on exertion
HF: Heart failure
HLP: Hyperlipidemia
HRCT: High-Resolution Computed Tomography
HTN: Hypertension
MACE: Major adverse cardiovascular events
MI: Myocardial infarction
PCR: Polymerase chain reaction
PCI: Percutaneous coronary intervention
PND: Paroxysmal nocturnal dyspnea
WHO: World Health Organization
